# Meta4: a web application for sharing and annotating metagenomic gene predictions using web services

**DOI:** 10.3389/fgene.2013.00168

**Published:** 2013-09-05

**Authors:** Emily J. Richardson, Franck Escalettes, Ian Fotheringham, Robert J. Wallace, Mick Watson

**Affiliations:** ^1^ARK-Genomics, The Roslin Institute and R(D)SVS, University of EdinburghEaster Bush, Midlothian, UK; ^2^Ingenza Ltd., Roslin BioCentreMidlothian, UK; ^3^Rowett Institute of Nutrition and Health, University of AberdeenAberdeen, UK; ^4^Edinburgh Genomics, University of EdinburghEdinburgh, UK

**Keywords:** metagenomics, database, web service, gene discovery, bioinformatics

## Abstract

Whole-genome shotgun metagenomics experiments produce DNA sequence data from entire ecosystems, and provide a huge amount of novel information. Gene discovery projects require up-to-date information about sequence homology and domain structure for millions of predicted proteins to be presented in a simple, easy-to-use system. There is a lack of simple, open, flexible tools that allow the rapid sharing of metagenomics datasets with collaborators in a format they can easily interrogate. We present Meta4, a flexible and extensible web application that can be used to share and annotate metagenomic gene predictions. Proteins and predicted domains are stored in a simple relational database, with a dynamic front-end which displays the results in an internet browser. Web services are used to provide up-to-date information about the proteins from homology searches against public databases. Information about Meta4 can be found on the project website^1^, code is available on Github^2^, a cloud image is available, and an example implementation can be seen at

## INTRODUCTION

Whole-genome shotgun (WGS) metagenomics can be defined as the application of high-throughput sequencing technologies to whole environmental samples, enabling scientists to assay the genomes of all organisms within a particular ecosystem, be it the human gut microbiome (Yatsunenko et al., 2012), permafrost (Mackelprang et al., 2011), or the Sargasso Sea (Venter et al., 2004). One of the aims of such endeavors is to discover novel enzymes that may have be of use to the biotechnology industry (Cowan et al., 2005), and metagenomics has been identified as a major mechanism for increasing the “sequencing space” from which to discover new biocatalysts (Cowan et al., 2004).

Whole-genome shotgun metagenomics experiments routinely produce hundreds of gigabases of sequencing data. A generalized analysis pipeline for such data is to (i) assemble the genomic data *de novo*; (ii) predict genes and proteins on the resulting contigs and scaffolds; (iii) assign domains and function to those proteins; (iv) interpret those findings within the biological context. It is not unusual for such studies to generate several million novel genes/proteins – Venter et al. (2004) reported over 1.2 million novel genes, and Hess et al. (2011) reported over 2.5 million putative genes, 27755 containing a domain of interest: those relevant to biomass degradation.

Metagenomic assembly poses specific problems over and above those of single genome assembly. The attempt to simultaneously assemble thousands of different genomes often results in large and complex assembly graphs. These require more memory to create and query, and also often require extra information in order to find true paths through the graphs. Ray Meta (Boisvert et al., 2012) is a massively distributed metagenome assembler that uses message passing, whereas Pell et al. (2012) reduce memory requirements using a bloom filter and use kmer connectivity to improve the assembly process. Other tools attempt to partition the assembly graph – Meta IDBA using graph connectivity (Peng et al., 2011) and MetaVelvet using both coverage and connectivity (Namiki et al., 2012). Finally, MetAMOS (Treangen et al., 2013) is a metagenomics pipeline that combines a number of published tools for metagenomic analysis.

Once the raw metagenomic reads have been assembled into contigs and scaffolds, the next stage is an attempt to predict the location of genes. Here again, metagenomics poses particular problems when compared to single bacterial genome annotation (recently reviewed in Richardson and Watson, 2013). Specifically, traditional bacterial gene predictors use models trained on a single, related genome; as with metagenomics we sequence thousands of genomes simultaneously, this is no longer appropriate. A number of tools have been published for metagenomic gene prediction, including MetaGeneAnnotator (Noguchi et al., 2008), Orphelia (Hoff et al., 2009), FragGeneScan (Rho et al., 2010), and Glimmer-MG (Kelley et al., 2012). Yok and Rosen (2011) propose a combination of tools.

Once genes have been annotated, domains can be assigned to protein-coding genes using traditional approaches, such as HMMER (Eddy, 2009) searches of domain databases such as Pfam (Punta et al., 2012), and the use of tools such as InterProScan (Mulder and Apweiler, 2007).

After raw reads from metagenomics experiments have been assembled and annotated, researchers are left with a very large and rich dataset which can be difficult to query and share. Tools that allow multiple users to browse and query such datasets, either privately within a consortium, or as part of a public collaboration, remain under-developed. It is essential that simple, open, and flexible tools are provided to allow scientists to easily access the outputs of metagenomic gene discovery projects. Here we describe Meta4, a web application that is easy to install, that should work on any standard LAMP (Linux, Apache, MySQL, PHP) server, and which allows users to search and browse large collections of metagenomic gene predictions in a user-friendly web interface. In addition, Meta4 makes use of web services to provide up-to-date annotation.

There are a few existing tools for organizing and analyzing metagenomic data on the web; however, despite being feature-rich, many are closed systems. The integrated microbial genomes and metagenomes (IMG/M) system (Markowitz et al., 2012) allows comprehensive analysis of genomes and metagenomes sequenced at the Joint Genome Institute (JGI). However, the system is not open-source, it is not possible to download the code and create a local installation, the software is only extensible by the authors and it is not easy to integrate your own data – one must e-mail the authors and request integration. Similarly, the Community cyberinfrastructure for Advanced Microbial Ecology Research and Analysis (CAMERA; Sun et al., 2011) is a workflow-based, feature-rich website for metagenomic analysis; however, the same issues remain in that it is not open-source, it is only extensible by the authors, it is not possible to create a local installation, and users must e-mail the authors to request integration of their data. Luckily, the metagenomics RAST server (MG-RAST; Meyer et al., 2008), a very popular and comprehensive tool for metagenomic data analysis, is far more open, with users encouraged to submit their own data, and the code is available on github^3^. However, even the authors admit, local installations of the tool are difficult, they advise against it, and no support for such an undertaking is available^4^.

All three tools are feature- and function-rich, and aim to be complete systems for the assembly, annotation, and comparison of multiple metagenomic samples. One problem with systems such as IMG/M and CAMERA is an inability for users to maintain data privacy; once data is uploaded to these systems, it is available for the public to see. MG-RAST does have the option to submit to a private queue, but this is a low priority queue. As such, these tools are not designed for the simple task of sharing large amounts of data quickly and simply. Meta4 is not designed to compete with these tools in terms of functionality; rather, it is a simple tool allowing the rapid sharing of metagenomic results that is easily extensible by the addition of web services. It is possible to set up a Meta4 database in less than 30 min on a simple Linux server such as an Amazon EC2 micro instance. Meta4 is a lightweight tool, completely open-source, easy to install locally and easy to add additional functionality through web services.

Meta4 was developed on an Amazon EC2 micro instance using a CloudBioLinux (Afgan et al., 2012) image. All code is available via Github. An example Meta4 database can be queried at  containing an assembly of the Hess et al. (2011) data.

## MATERIALS AND METHODS

**FIGURE 1:**
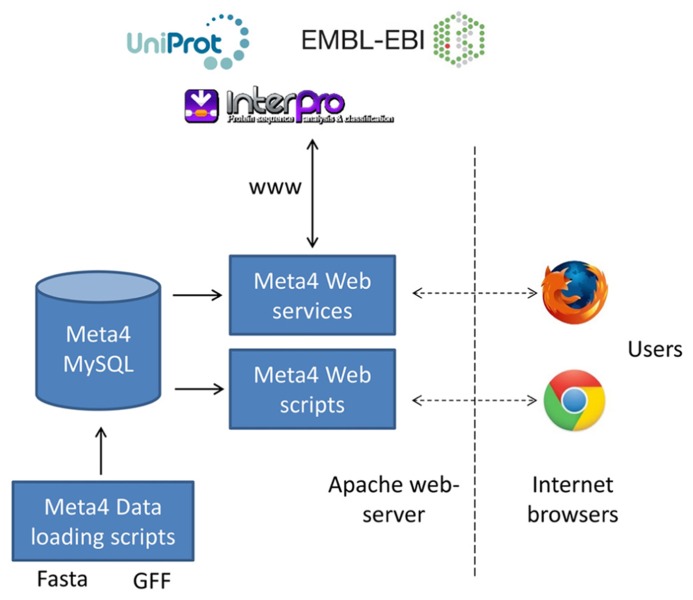
**The overall structure of Meta4, which shows the relationship between the MySQL database, the data loading scripts, the web interface, external web services, and the users**.

The overall structure of Meta4 is shown in **Figure 1**. Central to the system is the Meta4 MySQL database, which stores information on samples, assemblies, gene predictions, and protein domain information. The choice to store some basic annotation in the database itself allows users to query the available gene predictions on domains of interest. Without such annotation, it would be very difficult for users to filter the large numbers of gene predictions in metagenomic datasets. We have chosen to store information on protein domains, rather than the results of homology searches (e.g., BLAST), as often domain searches are more sensitive to distant homology. Information can be loaded into the database from common formats using the database loading scripts, including GFF3 (gene predictions) and fasta (contigs and scaffolds). A web form is provided that allows users to query the database and information is presented in two ways: firstly, data extracted directly from the Meta4 database is presented in the browser; secondly, data extracted from the Meta4 database is provided to a range of web services, and the results of those web services presented in the browser. This allows for the latest, live, up-to-date annotation to be displayed for each gene prediction, and is a key feature of Meta4.

### INTERFACE AND WEB SERVICES

The dynamic web interface is written in Perl/CGI and should run on any apache web-server with minimal setup. The user is presented with a form including several parameters for search and retrieval of genes/proteins within the database. The results are returned as an HTML table, and consist of two parts – those that return information stored in the database, and those returned from web services.

We have implemented three web services in Meta4. The first uses the EBI’s SOAP wublast interface (McWilliam et al., 2009), querying Uniprot (Magrane and Consortium, 2011) with a protein sequence retrieved from the database. The top 10 results are returned and these represent the most up-to-date homology information for that protein within Uniprot.

The second uses the Uniprot REST web service (Jain et al., 2009). Domains associated with a particular protein are extracted from the database and used as input to search Uniprot. In this way, known proteins with a similar domain structure to that being queried are returned and presented to the user. Users are then able to see the protein name and species of similar proteins, and can click through to the Uniprot entry.

The third uses the EBI’s InterproScan (Mulder and Apweiler, 2007) SOAP interface (McWilliam et al., 2009), querying up to 14 separate protein domain databases with a protein sequence retrieved from the database. The image and text returned also represent the most up-to-date information publicly available for the domains predicted within the query protein.

### DATABASE STRUCTURE

The Meta4 MySQL database models the following specific entities and their relationships:

(i) Sample: information about a specific biological sample that has been sequenced. In reality we imagine most researchers will store this information in some other database [e.g., a laboratory information management system (LIMS)], but this table allows metagenomic data to be linked to specific samples.
(ii) Assembly: information about a *de novo* assembly of data from a biological sample. This allows for multiple different assemblies of the same sample. The parameters of the assembly can be stored as tag = value pairs in an assembly_param table.
(iii) Contig: models the contigs that are output as the result of an assembly. We do not explicitly differentiate between contigs and scaffolds. In this instance, a contig simply describes a single, contiguous sequence obtained from a metagenomic assembly.
(iv) Gene prediction: information on the genes predicted on any given contig, including the location on the contig, and the DNA and protein sequence.
(v) Domain database: contains information on the domain database used and allows each gene prediction to have hits to multiple domain databases [e.g., PROSITE (Sigrist et al., 2010) and Pfam (Punta et al., 2012)] or multiple versions of the same domain database.
(vi) Protein domain: information on the domains within each domain database.
(vii) Domain match: storage of the link between gene predictions and protein domains, including location of the match, bit score and e-value.

Crucially, this structure allows multiple assemblies of the same biological sample, as it is common to carry out multiple genome assemblies using different software and parameter sets (which can be flexibly stored in the assembly_param table). Domain matches from multiple databases may also be stored.

### CODE STRUCTURE AND DEVELOPMENT

We have implemented the Meta4 data model in MySQL with an interface written in Perl and Perl CGI. The code has been tested on CloudBioLinux (Afgan et al., 2012) and a local Scientific Linux server, and should work on any standard LAMP server. The github repository contains the following folders:

(i) sql: SQL for creating the MySQL database.
(ii) examples: example files used to create a simple instance of Meta4.
(iii) scripts: perl scripts to load information and data into a Meta4 database.
(iv) cgi_scripts: perl CGI scripts that provide an interface to query the data within a Meta4 database.

A README file is included in the distribution which gives accurate instructions on how to create a Meta4 database that is accessible via a web browser. If the import scripts are run with no parameters, simple instructions are printed to the terminal.

Meta4 is released under an open-source license and we welcome active participation in the project. Whilst Meta4 is suitable for release and publication in its current form, there are many ways in which Meta4 could be developed. For example, currently users must import data using Linux command-line scripts, rather than a graphical user interface (GUI); also, we present scripts to import data from the output of pfam_scan.pl^5^, and we welcome contributions that are able to import data from other software formats.

## RESULTS

### EXAMPLE DATASET

We have created an example Meta4 database and the results can be browsed at . Briefly, we downloaded data from Hess et al. (2011) (SRA accession SRA023560) and assembled the reads using SOAPdenovo (Li et al., 2010). Open-reading frames greater than 200 bp in length were extracted as putative genes. Pfam-A domains were annotated using pfam_scan.pl^5^. As the experiment was designed to find novel biomass degrading genes, we encourage users to enter “glyco_hydro” into the “Name” field and click “Submit.”

### BROWSING GENE PREDICTIONS

**FIGURE 2:**
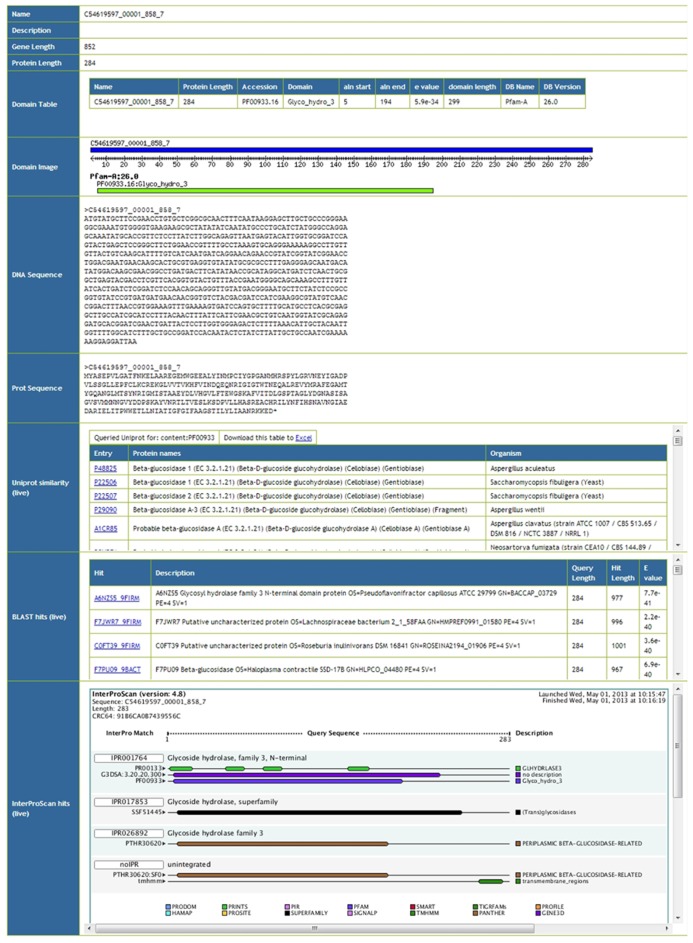
**Screenshot of the Meta4 results interface, showing information extracted from the Meta4 database, and information from web services (marked as “live” in the table)**.

Meta4 allows users to browse information on particular gene predictions. An example screenshot of such information can be seen in **Figure 2**. Basic information such as the gene name, description, and sequence lengths are extracted from the database. Protein domains annotated within the database are also extracted, and presented as both a table and an image. Furthermore, the actual gene and protein sequences are presented, and formatted correctly. Afterward, live information is presented from the three web services. Firstly, proteins with the same domain structure are extracted from Uniprot, and presented as a table. Secondly, the top 10 BLAST hits against Uniprot/TREMBL are presented. In this way, users are able to see similar proteins in Unprot by domain structure and by sequence homology, and can click through to the relevant entries. Finally, results from the InterProScan web service are presented, both as an image and as text. As InterProScan searches 14 different domain databases, we are able to view more information here than the simple domain information stored in the Meta4 database. A key advantage of Meta4 is that information and annotation about the protein in question is served to the user in real time, and therefore represents the most up-to-date information possible.

### WEB INTERFACE

The web interface has been tested on Firefox (Windows, Linux, Android), Safari (Windows, Mac), Opera (Windows, Android), Konqueror (Linux), Chrome (Windows), the Android native browser, and Internet Explorer (Windows). All features work on all browsers, except Internet Explorer 8 (Windows). Our implementation of the EBI’s InterproScan web service produces an in-line image using the data URI (uniform resource identifier) scheme, and we understand Internet Explorer 8 to have a 32 Kb limit for these. This is fixed in Internet Explorer version 9.

### AMAZON EC2 CLOUD IMAGE

An Amazon Machine Image (AMI) is available (EU-WEST: ami-46687f32). The AMI is based on Ubuntu Precise 12.04 (64 Bit) with additional dependencies installed, including Meta4. We have loaded the example data packaged with Meta4, and the system is available from the cgi-bin of the installed Apache2 web-server. Full instructions on how this was set up are available here: 

## DISCUSSION

The role of Meta4 is to allow bioinformaticians to share the results of metagenomic assembly and annotation with collaborators, and to provide those collaborators with a simple web-based interface with which to query and browse the data. It is not intended to compete with tools that aim to assemble, annotate, and functionally or taxonomically compare multiple metagenomic datasets; rather, it is a simple web application that can be used to search and browse large amounts of information quickly, and retrieve genes and proteins that may be of interest for further studies.

The key advantages of Meta4 are:

(i) Simplicity: Meta4 is incredibly simple and can be installed in minutes on a standard LAMP server, either using the git repository or by using the Amazon EC2 image. A new Meta4 instance can be created rapidly from standard formats using the scripts provided. In addition, Meta4 is completely open-source.
(ii) Use of web services: by using web services, Meta4 ensures the latest annotation results are delivered to users. In contrast, other systems store pre-computed results which can rapidly become out-of-date. By using web services, it is easy to extend the functionality of Meta4.
(iii) Separation of data delivery from data analysis: existing web-based systems combine assembly and annotation with results presentation. By separating the search/browse function from data analysis, Meta4 allows bioinformaticians to use an assembly and annotation pipeline of their choice, and still share their results with collaborators through a user-friendly web interface.
(iv) Access control: often when one submits data to a public web-server, a commitment is made to make the data publicly available. Meta4 can be set up on a private intranet in minutes, ensuring data privacy; alternatively, cloud Meta4 instances can be limited to specific IP addresses. Thus Meta4 allows both public and private sharing of data.

Managing the large amounts of data from WGS metagenomics projects is a challenge and there is a need for simple tools that enable scientists to access and query the results. We present Meta4, a simple database for the storage of proteins and their domains predicted from metagenomics experiments. Meta4 is lightweight, easy to install and deploy, and can handle large amounts of data. The system presents information to scientists in a format they understand via a web interface. Meta4 is easily extensible through the addition of web services, and despite not being as feature-rich as some existing systems, benefits from being open-source, lightweight and easy to install and deploy. The use of web services means that the data served to users is as up-to-date as the underlying primary database, which is an advantage over large data warehouses whose data may become out-of-sync with the primary data source. Meta4 is available under an open-source license at .

Despite the increasing number of published algorithms for metagenomic assembly and annotation, the complexity of the problem is such that errors are common. Attempts must be made to assess the quality of metagenomic assemblies prior to annotation, especially to ensure inappropriate joins are not made during the contig and scaffold production steps. Metagenomic assemblies are often highly fragmented, and this can affect gene prediction and protein domain annotation. Once specific protein targets have been identified from metagenomic datasets, we recommend a manual annotation step to ensure the gene location (start and end) and protein domain structures are correctly defined.

## Conflict of Interest Statement

The authors declare that the research was conducted in the absence of any commercial or financial relationships that could be construed as a potential conflict of interest.

## Footnotes

3

4

5
